# Long Noncoding RNA DSCAM-AS1 Facilitates Colorectal Cancer Cell Proliferation and Migration via miR-137/Notch1 Axis

**DOI:** 10.7150/jca.46562

**Published:** 2020-09-23

**Authors:** Jing Xu, Guanghai Wu, Yongjie Zhao, Youkui Han, Shuai Zhang, Chao Li, Judong Zhang

**Affiliations:** Department of General Surgery, Tianjin Union Medical Center, Jieyuan Road 190, Hongqiao District, Tianjin, 300121, PR China

**Keywords:** Long noncoding RNAs, MicroRNAs, Colorectal cancer, Proliferation, Migration

## Abstract

Growing evidences demonstrate that long noncoding RNAs (lncRNAs) participate in various cancers including colorectal cancer (CRC). In the current study, we found that the expression of DSCAM-AS1 in CRC tissues and cell lines was significantly upregulated, and was positively correlated with metastasis status and advanced stage of CRC. In addition, Kaplan-Meier assays also indicated that the expression of DSCAM-AS1 was correlated with poor prognosis in patients with CRC. Silence of DSCAM-AS1 inhibited proliferation and migration of CRC cells. Subcellular fractionation and FISH analyses suggested that DSCAM-AS1 was majorly distributed in cytoplasm of HT29 and LOVO cells. Thus, DSCAM-AS1 might act as a competing endogenous RNA (ceRNA). Subsequently, RT-qPCR results displayed that the expression of miR-137 in CRC tissues was relatively lower than that in the neighboring normal tissues. The interaction between miR-137 and DSCAM-AS1 was demonstrated by luciferase reporter assay. Functionally, miR-137 reversed the pro-proliferation and -metastasis effect of DSCAM-AS1 on CRC cells. Collectively, DSCAM-AS1 promotes CRC progression via sponging miR-137. MiR-137 can suppress the expression of Notch-1, a novel signaling regulating cell proliferation and EMT, by working on the 3'UTR of Notch-1. At last, Notch-1 overexpression or miR-137 inhibition could restore the DSCAM-AS1 silencing-mediated repressive function on cell proliferation and migration. The above data suggested that, DSCAM-AS1 may contribute to CRC cell proliferation and migration by targeting miR-137/Notch-1 axis.

## Introduction

Colorectal cancer (CRC) is the third most commonly diagnosed cancer worldwide and is one of the leading causes of cancer-specific death 1, with more than 1.1 million cancer deaths expected by 2030 2. Evidences showed that the death rate of CRC is still about 35% in spite of great improvement of systematic and comprehensive treatment was achieved 3. However, there are still more than 50% patients with CRC died from distant metastasis 4. Many efforts have been devoted to shedding light upon the molecular mechanisms of CRC. However, the related molecular mechanisms are elusive and remain unknown. Elucidation of the mechanisms involved in tumor metastasis and progression may help of the effective diagnosis and treatments, which will further improve the clinical outcome of patients with CRC.

Long noncoding RNAs (lncRNAs) are important components of noncoding RNA family. They are RNA transcripts with over 200 nucleotides in length 5. Accumulating evidences have shown that lncRNAs exhibit extensive regulatory functions in several biological processes, such as DNA damage and repair, vasculo-genesis, miRNAs silencing and apoptosis 6. Recent studies show that lncRNAs are closely associated with various human cancers 7, by regulating proliferation, differentiation and metastasis of cancer cells 8. LncRNA-PCAT6 promotes proliferation, migration, and invasion of osteosarcoma via activation of TGF-β pathway by sponging miR-185-5p 9.

LncRNAs act as competing endogenous RNAs (ceRNAs), thereby modulating the expression of different microRNAs (miRNAs) in a cell-type dependent manner 10. The binding of miRNAs to lncRNAs decreases miRNA levels and leads to the increase in the expression of miRNA target genes 11, 12. LncRNA DSCAM-AS1, a cancer-related lncRNA, has been reported to be dysregulated in various types of human cancers 13, 14. Recent studies showed that lncRNA DSCAM-AS1 indicates poor prognosis of ovarian cancer 15, luminal breast cancer 16, and melanoma 17. However, the specific role of DSCAM-AS1 in CRC has not been clarified.

In this current study, we reported that the level of DSCAM-AS1 was upregulated in CRC tissues relative to that in corresponding adjacent normal tissues. Knockdown of DSCAM-AS1 suppressed the proliferation and migration of CRC cells. The results delineate that DSCAM-AS1 promotes CRC cell proliferation and migration through the regulation of miR-137/Notch1 axis. Therefore, our study will provide new insights into the molecular function of the DSCAM-AS1/miR-137/Notch1 axis in the pathogenesis of CRC and highlight the potential of lncRNAs to act as new therapeutic targets in CRC.

## Methods

### Sample collection

Fifty-one CRC tissues and adjacent normal tissues were gained from Tianjin Union Medical Center from August 2017 to January 2019. Patients were eligible for the study if (1) they did not receive any chemotherapy or radiotherapy prior to surgery; (2) complete medical records including patient demographics, clinical data and follow-up information were available; (3) surgical specimens of tumor lesions were available for analysis. The clinical stage of these patients was determined according to the Tumor, Node, Metastasis (TNM) staging classification of the International Union against Cancer (UICC). This study was approved by Tianjin Union Medical Center. Written informed consents were obtained from patients. All specimens were immediately frozen using liquid nitrogen for the following experiments.

### Cell culture and cell transfection

Four human CRC cell lines (HT29, LOVO, SW480 and PKO) and a normal human colon epithelial cell line NCM460 were purchased from the cell bank of the Chinese Academy of Sciences (Shanghai, China). Cells were cultured in RPMI1640 medium (Gibco, Carlsbad, CA, USA), and were supplemented with 10% fetal bovine serum (FBS, Gibco, NY, USA). All cell lines were cultured at a condition of 37°C with a humidified atmosphere containing 5% CO_2_ 18. Specific shRNAs against DSCAM-AS1 (sh-DSCAM-AS1#1 and sh-DSCAM-AS1#2) and corresponding NCs (sh-NCs), together with the pcDNA3.1 vector targeting Notch-1 and the empty vector, were acquired from RiboBio (Guangzhou, China). Moreover, miR-137 mimics, miR-137 inhibitors, NC mimics and NC inhibitors were acquired from GenePharma (Shanghai, China). HT29 or LOVO cells were transfected with these plasmids through Lipofectamine 2000 (Invitrogen, CA, USA), separately.

### RT-qPCR

Total RNA was extracted using TRIzol reagent (Invitrogen) and inversely transcribed into cDNA using a Reverse Transcription Kit (Thermo, USA). Then RT-qPCR was carried out using SYBR Select Master Mix (Thermo Fisher Scientific, Waltham, MA, USA) or the TaqMan MicroRNA assay Kit (Thermo Fisher Scientific, Inc.). Relative expression was normalized to U6 or GAPDH and all were measured by the comparative Ct (ΔΔ Ct) method.

### CCK-8 assay

Cells were seeded at a density of 1x10^5^/ml into 96-well plates. After 24, 48, 72 and 96 hours incubation with different compounds as described above, 10ml CCK-8 reagents (Qianshang, Wuxi, China) was added to each well. The 450 nm absorbance of the plates was determined by a microplate reader.

### Transwell assay

Transwell assay was performed to examine the migration abilities of OS cells. The detailed method was described previously 19.

### Luciferase reporter assays

DSCAM-AS1-wt, DSCAM-AS1-mut, Notch-1-wt or Notch-1-mut sequence was inserted into pMIR-reporter (Promega, USA). Dual-Luciferase Reporter Assay Kit (Promega, Madison, WI, USA) was utilized to determine the luciferase activity. The detailed protocol was in accordance with the instruction of the manufacturer.

### Subcellular fractionation

Nuclear or cytoplasmic RNA in cells was separated as well as purified via a Cytoplasmic and Nuclear RNA Purification Kit (Norgen Biotek, Thorold, Canada). Expression patterns of DSCAM-AS1, GAPDH and U6 in nuclear and cytoplasm fractions were sequentially determined via RT-qPCR, respectively.

### Immunofluorescence (IF) assays

Cells in plates were fixed with 4% paraformaldehyde for 30 min and permeated with 0.3% Triton X-100 for 5 min at room temperature. Then, cells were blocked with 5% in bovine serum album in phosphate-buffered saline for 2 h at room temperature and incubated with primary antibodies for anti-E-cadherin (1:1000, no. 3195, Cell Signaling Technology, USA), anti-vimentin (1:2000, no. 10366-1-AP, Proteintech, China) at 4 °C overnight. The NC group was incubated with 1% bovine serum album. After three times washes with phosphate-buffered saline, cells were incubated with secondary antibody. DAPI was added to the samples to visualize cell nuclei. The staining was captured by a Leica DMI4000 B automated inverted microscope equipped with a Leica DFC300 FX camera.

### Western blotting

Total protein in cells was extracted with a protein extraction reagent (Thermo, USA) according to the manufacturer's instructions. The proteins were transferred onto polyvinylidene fluoride membrane (Millipore, USA), and incubated overnight with primary antibodies. The primary antibodies and their dilutions used were as follows: Notch-1 (1:1000, Cell Signaling Technology, USA) and β-Action (1:1000, Proteintech, USA). After washing, the secondary antibody was used for detection. Proteins were visualized by ECL (Advansta, USA). Intensity of the bands was analyzed with Image J software.

### Statistical analysis

SPSS 19.0 software was used for statistical analysis. All the values were expressed as mean ± Standard Deviation (SD). Data was analyzed using one-way analysis of variance (ANOVA) or the *LSD*-test. Pearson correlation test was conducted to measure the association among DSCAM-AS1, miR-137 and Notch-1 expression. A value of *P* < 0.05 was considered statistically significant.

## Results

### The upregulation of DSCAM-AS1 is associated with poor prognosis in CRC

**Figure 1:**
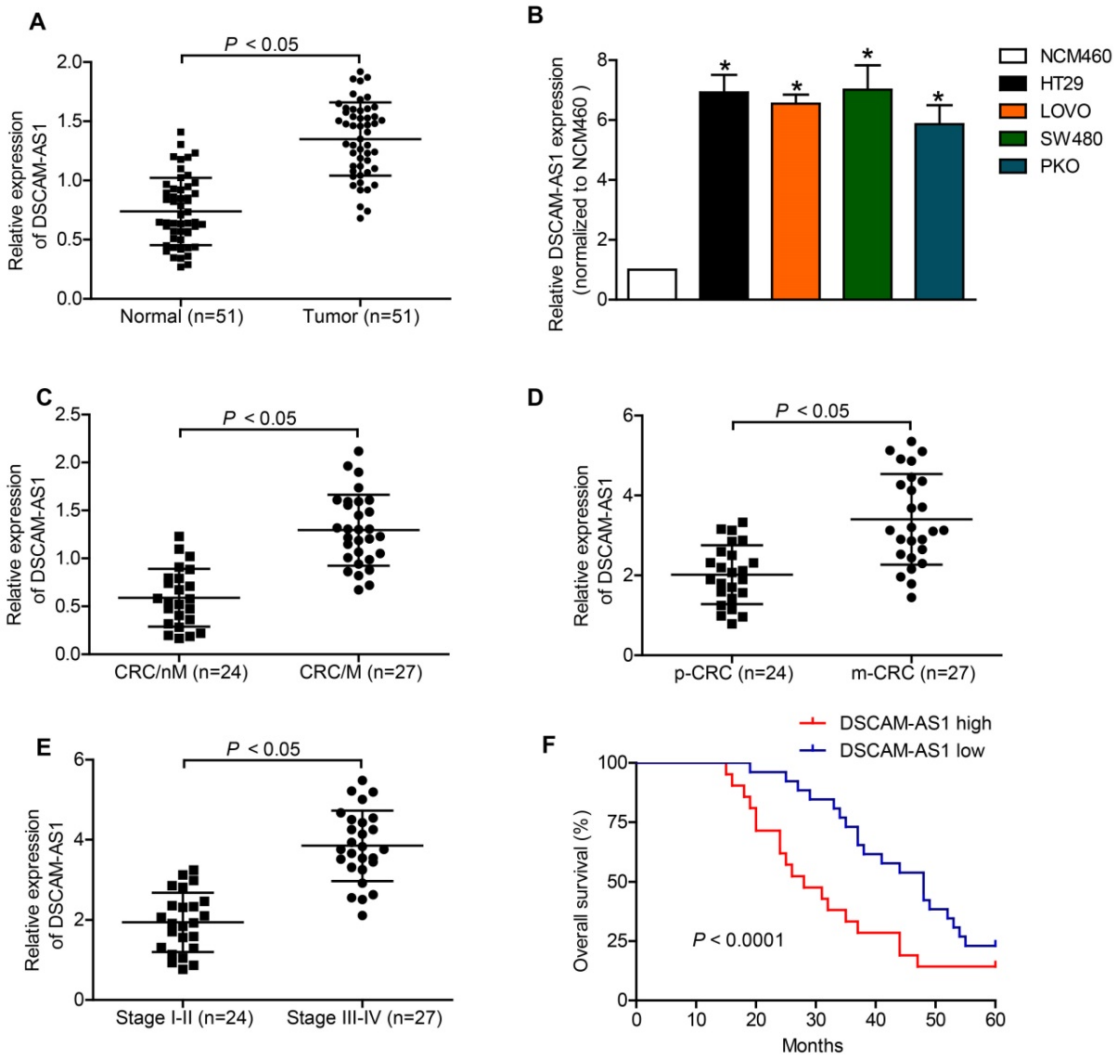
** Upregulation of DSCAM-AS1 is associated with poor prognosis in CRC.** A: DSCAM-AS1 expression was detected by RT-qPCR. B: DSCAM-AS1 was upregulated in four kinds of CRC cell lines HT29, LOVO, PKO and SW480, as comparing with that of a normal human colon epithelial cell line NCM460. *P < 0.05. C: RT-qPCR analysis of DSCAM-AS1 expression in CRC tissues without metastasis (CRC/nM) and CRC tissues with metastasis (CRC/M). D: RT-qPCR analysis of DSCAM-AS1 expression in primary CRC tissues (p-CRC) and CRC tissues from metastatic sites (m-CRC). E: RT-qPCR analysis of DSCAM-AS1 expression in stage I-II CRC tissues and stage III-IV CRC tissues. F: Kaplan-Meier survival analyses indicated that upregulation of DSCAM-AS1 in CRC patients predicted worse overall survival.

Existing evidence has delineated a conspicuous upregulation of DSCAM-AS1 in various types of human cancers 13, 14, 20, yet the expression status of it in CRC remains unknown. Herein, RT-qPCR was applied to measure the expression of DSCAM-AS1 in CRC tissue samples and CRC cells. In comparison with neighboring normal tissues, DSCAM-AS1 expression was significantly increased in CRC tissues (Fig 1A). Further analysis indicated that DSCAM-AS1 expression was upregulated in CRC cell lines (Fig 1B). We also found that the upregulation of DSCAM-AS1 was positively correlated with metastasis status and advanced stage of CRC (Fig 1C-E and Table 1). Then, Kaplan-Meier assays indicated that the patients with higher DSCAM-AS1 expression displayed poorer overall survival rates than those with lower expression. Collectively, DSCAM-AS1 expression was increased in CRC tissues and cell lines and correlated with poor prognosis in CRC patients.

### Downregulation of DSCAM-AS1 inhibited proliferation and migration of HT29 and LOVO cells

**Figure 2:**
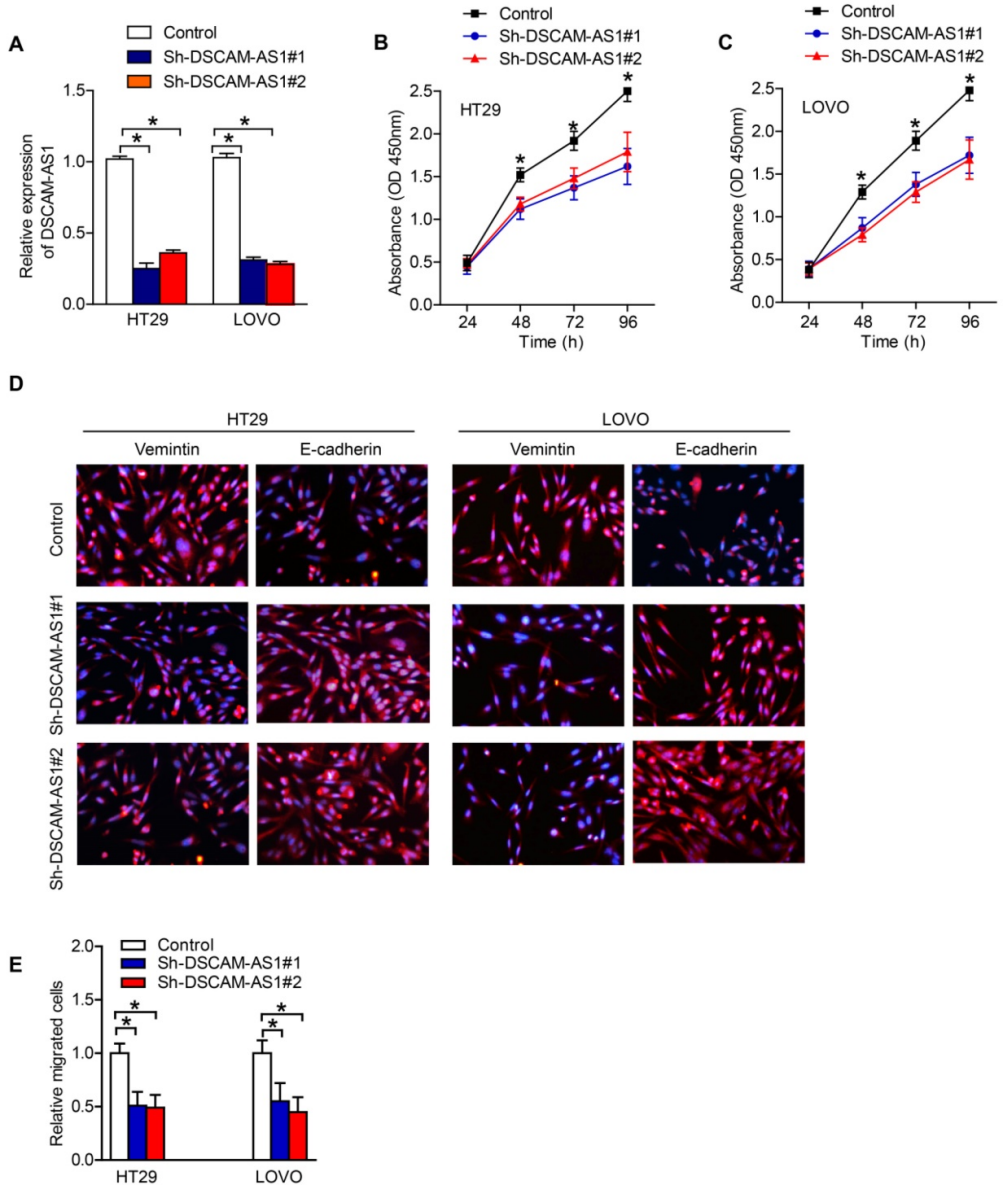
** DSCAM-AS1 promotes proliferation and migration of CRC cells.** A: RT-qPCR analysis of DSCAM-AS1 expression in the indicated cells. B-C: CCK8 was conducted to test cell proliferation in HT29 and LOVO cells. D: Immunofluorescence staining of vimentin and E-cadherin in HT29 and LOVO cells. E: Transwell assays were performed to examine the migration ability of HT29 and LOVO cells. *P < 0.05.

The above findings suggested that DSCAM-AS1 might act as a tumor promoter in CRC. Therefore, we firstly used specific DSCAM-AS1 siRNAs to knockdown DSCAM-AS1 in CRC cells. RT-qPCR showed that DSCAM-AS1 expression was knocked down in HT29 and LOVO cells successfully (Fig 2A). CCK-8 assay showed that knockdown of DSCAM-AS1 significantly inhibited the proliferation of HT29 and LOVO cells (Fig 2B and C). Epithelial to mesenchymal transition (EMT) is an important mechanism mediating the migration of cancer cells. IF was used to examine the change of expression of EMT related biomarkers. We examined the expression of epithelial biomarker E-cadherin and mesenchymal biomarker vimentin in CRC cells after transfection with sh-DSCAM-AS1. As our results showed, knocking down of DSCAM-AS1 alleviated EMT process in CRC cell lines (Fig 2D). In addition, experiments concerning cell migration analyzed the migration ability of HT29 and LOVO cells after DSCAM-AS1 was silenced. The data indicated that compared with negative control, deficiency of DSCAM-AS1 repressed cell migration (Fig 2E).

### DSCAM-AS1 sponges miR-137 in CRC cells

**Figure 3:**
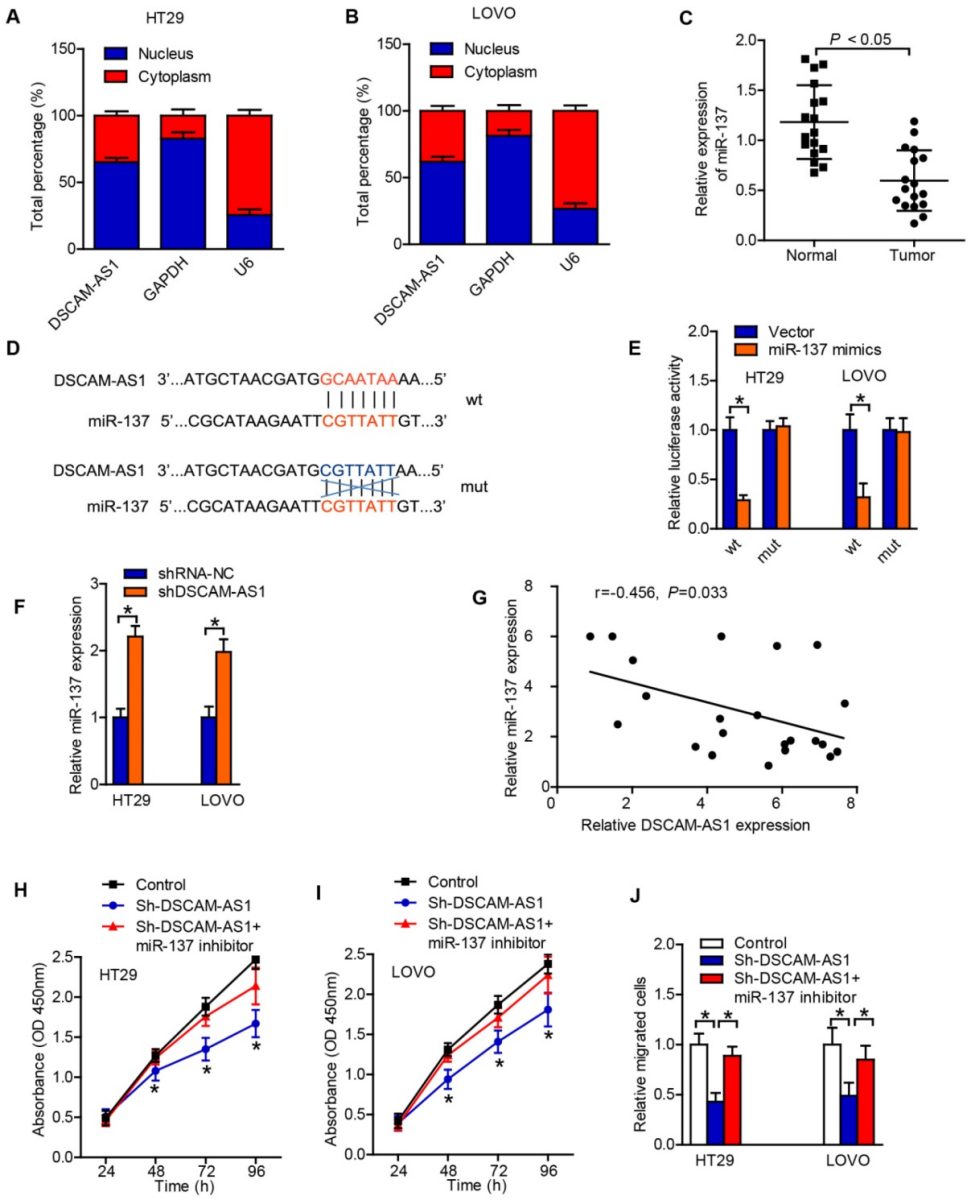
** DSCAM-AS1 sponges miR-137 in CRC cells.** A-B: Nuclear and cytoplasmic fractionation was analyzed for DSCAM-AS1 expression. C: The expressions of miR-137 in CRC tissues were detected by RT-qPCR. D: The potential binding sites between DSCAM-AS1 and miR-137. E: Luciferase reporter assay showed DSCAM-AS1-wt activity was impaired by miR-137. F: The expression of miR-137 in CRC cells was significantly upregulated when DSCAM-AS1 expression was downregulated identified by RT-qPCR. G: The expression of miR-137 was negatively correlated with DSCAM-AS1 expression in CRC tissues. H-I: CCK-8 was applied to test the effect of miR-137 on cell proliferation induced by DSCAM-AS1. J: Transwell assay was performed to analyze migration of HT29 and LOVO cells in the indicated groups. *P < 0.05.

With the intention of deciphering the potential mechanism of DSCAM-AS1 in CRC, we first adopted subcellular fractionation and FISH analyses to detect the localization of DSCAM-AS1 in cytoplasm and nucleus of CRC cells. The result suggested that DSCAM-AS1 was majorly distributed in cytoplasm of HT29 and LOVO cells (Fig 3A, B). Thus, DSCAM-AS1 might act as a ceRNA in tumor progression. Subsequently, the target of DSCAM-AS1 was predicted, and we found that miR-137 was a potential target of DSCAM-AS1. RT-qPCR results displayed that the expression of miR-137 in CRC tissues was relatively lower than that in the neighboring normal tissues (Fig 3C). According to the miRcode database, there was some potential binding sites between DSCAM-AS1 and miR-137, and hence miR-137 was chosen for the subsequent experiments (Fig 3D). The interaction between miR-137 and DSCAM-AS1 was examined by luciferase reporter assay and the results demonstrated that miR-137 decreased DSCAM-AS1-wt reporter's luciferase activity, but have no effect on DSCAM-AS1-mut reporter's luciferase activity (Fig 3E). In addition, the expression of miR-137 in CRC cells was significantly upregulated when the expression of DSCAM-AS1 was downregulated (Fig 3F). We also realized that the expression of DSCAM-AS1 was also negatively correlated with miR-137 expression in CRC tissues (Fig 3G). Functionally, miR-137 reversed the pro-proliferation and -metastasis effect of DSCAM-AS1 on CRC cells (Fig 3H-J). Collectively, DSCAM-AS1 may promote CRC progression via sponging miR-137.

### DSCAM-AS1 regulates miR-137/ Notch-1 axis

**Figure 4:**
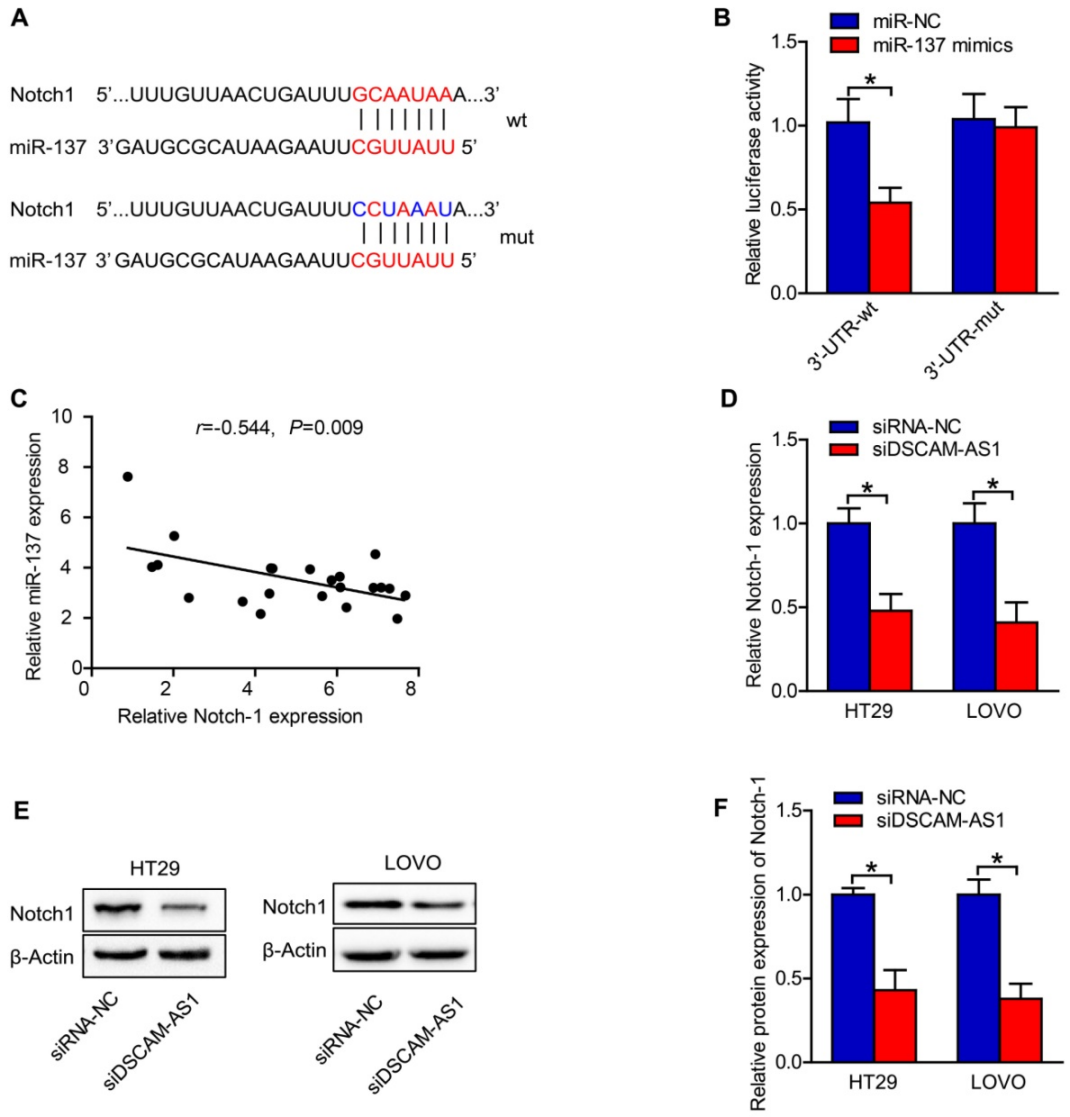
** DSCAM-AS1 regulates miR-137/Notch-1 axis.** A: The binding sites of miR-137 on 3′-UTR of Notch-1 were predicted. B: MiR-137 could regulate the luciferase activity of HT29 cells by binding to 3′-UTR of Notch-1 confirmed by the dual-luciferase reporter assay. C: MiR-137 expression was negatively correlated to the expression of Notch-1 in CRC tissues. D: RT-qPCR results showed that the expression of Notch-1 in CRC cells was significantly downregulated after transfection with sh-DSCAM-AS1. E: Western blot images of Notch-1 in CRC cells. F: Quantification of protein expression of Notch-1. *P < 0.05.

To explore the mechanisms underlying the role of miR-137 in CRC, we searched the putative targets for miR-137 using TargetScan, a miRNA target analyzing database, and found Notch1, which are known to be involved in cancer, as miR-137 targets. Notch-1 was associated with cell proliferation and EMT 21, 22. The binding sites of miR-137 on the 3′-UTR of Notch-1 were predicted by TargetScan (Fig 4A). Luciferase reporter assay was conducted to test whether Notch1 is a direct target of miR-137. We constructed reporters carrying either the wild type 3' UTR of human Notch-1 or a mutant one (Fig 4A). After co-transfecting with synthetic miR-137 mimics, the wild type reporter exhibited reduced luciferase activity, while the mutant one did not (Fig 4B). In addition, miR-137 expression was identified to be negatively correlated to the expression of Notch-1 in CRC tissues (Fig 4C). The RT-qPCR and western blotting results also revealed that the expression of Notch-1 in CRC cells was dramatically downregulated after transfection with sh-DSCAM-AS1 (Fig 4D-F). The above results suggested that DSCAM-AS1 acted as a sponge of miR-137 and regulated Notch-1 indirectly.

### DSCAM-AS1 contributes to CRC cell proliferation and migration by targeting miR-137/Notch-1 axis

**Figure 5:**
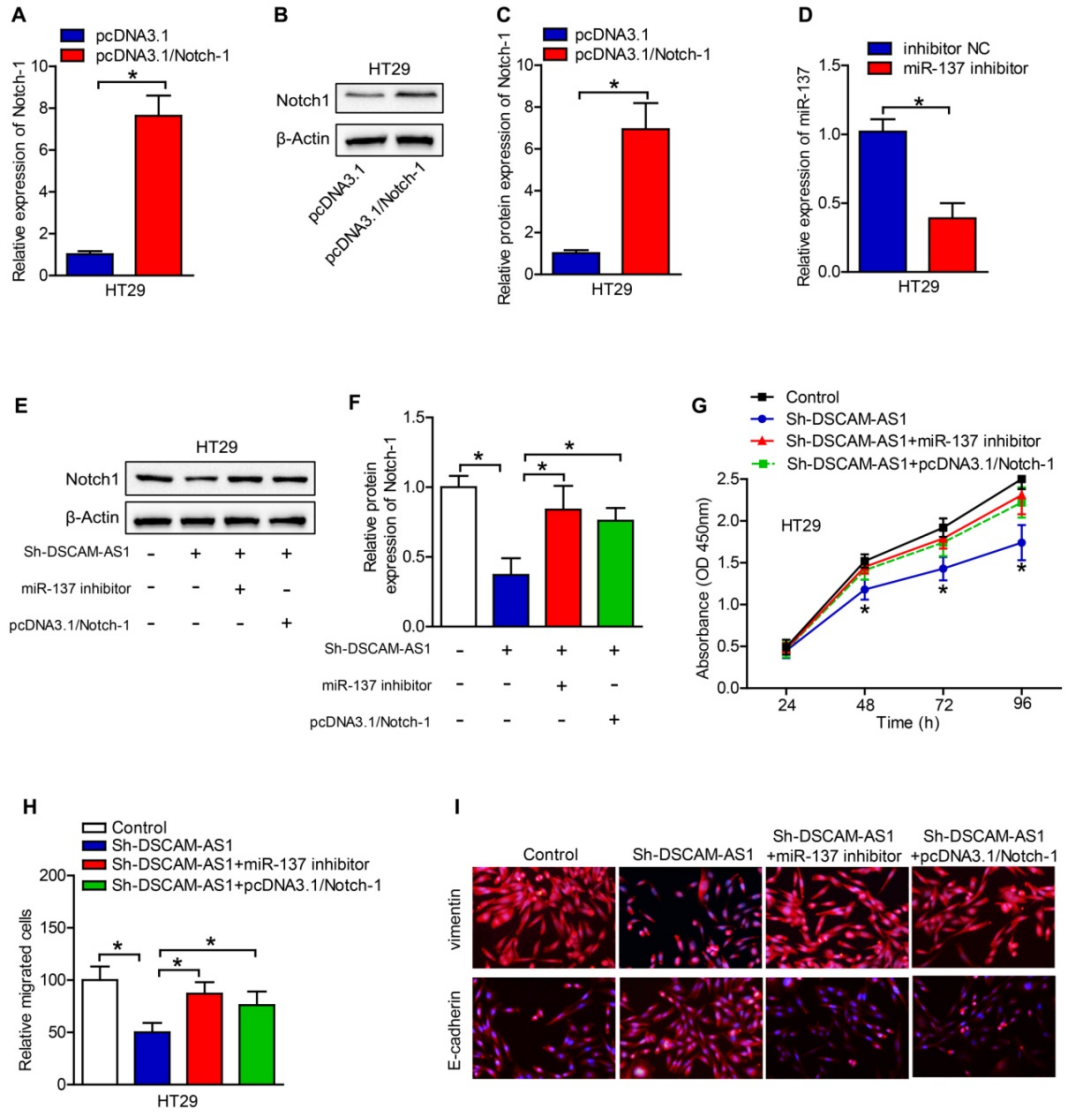
** DSCAM-AS1 contributes to CRC cell proliferation and migration by targeting miR-137/Notch-1 axis.** A-C: The efficiency of Notch-1 overexpression was analyzed by RT-qPCR and western blotting. D: The efficiency of miR-137 inhibition was analyzed by RT-qPCR. E: Western blotting was applied to test the protein expression of Notch-1. F: Quantification of protein expression of Notch-1. G: Cell proliferation assay (CCK-8) was used for the examination of cell proliferation ability in CRC cells transfected with different plasmids. H: The migration ability of transfected cells was measured by transwell assay. I: Immunofluorescence staining of vimentin and E-cadherin in HT29 cells. *P < 0.05.

Rescued-function test was used to clarify the DSCAM-AS1/miR-137/Notch-1 role in CRC. Prior to it, the efficiency of Notch-1 overexpression and miR-137 inhibition was analyzed by RT-qPCR, which turned out to be satisfactory (Fig 5A-D). Western blotting showed that miR-137 inhibition or Notch-1 overexpression could restore the DSCAM-AS1 silencing-mediated suppression on Notch-1 expression (Fig 5E, F). Then cell proliferation assay delineated that miR-137 inhibition or Notch-1 overexpression could restore the DSCAM-AS1 silencing-mediated repressive function on cell proliferation (Fig 5G). Cell migration assays confirmed that the attenuated migration ability of CRC cells caused by DSCAM-AS1 deficiency could be reversed by Notch-1 upregulation or miR-137 suppression (Fig 5H, I). Taken together, DSCAM-AS1 elicits promoting impact on CRC cell proliferation and migration via miR-137/Notch-1 axis.

## Discussion

The identification of sensitive biomarkers for prognosis and diagnosis and novel therapeutic targets has a very essential clinical significance for the improvement of long-term survival of CRC. In recent years, growing evidence has suggested that lncRNAs are tightly linked to the initiation and development of human cancers 13, 14, 20, 23. Previous studies have suggested the vital role of DSCAM-AS1 in hepatocellular carcinoma 24, breast cancer 20 and ovarian cancer 15. Recently, it was also reported that DSCAM-AS1 might promote the progression of colorectal cancer 25. However, the specific role of DSCAM-AS1 in CRC has not been fully elaborated.

This current study explored the role of DSCAM-AS1 in CRC and the underlying mechanisms. In this study, the expression of DSCAM-AS1 in CRC tissues and CRC cell lines was significantly elevated, and was positively correlated with metastasis status and advanced stage of CRC. In addition, the results of Kaplan-Meier assays also indicated that the expression of DSCAM-AS1 was correlated with poor prognosis in patients with CRC. Silence of DSCAM-AS1 inhibited proliferation and migration of CRC cells. Briefly, DSCAM-AS1 is a cancer-promoting gene in CRC. After confirming the proliferation and migration promotion role of DSCAM-AS1 in CRC, we further exploring the relating mechanisms. DSCAM-AS1 could serve as a sponge for miR-137 to upregulate Notch-1 expression, thus promote proliferation and migration of CRC cells. Overall, our findings revealed that DSCAM-AS1 could serve as a novel prognostic biomarker and therapeutic target in CRC.

The ceRNA hypothesis was proposed to describe the function of many lncRNAs as ceRNA to protect the genuine targets of miRNAs from silencing or translational suppression 26. LncRNA-miRNA-mRNA axis has been reported to play important roles in CRC 27-29. Although the expression and function of DSCAM-AS1 have been reported in previous studies in several tumors, the potential mechanisms involved have not been clarified. By predicting with miRcode, we found that miR-137, a novel microRNA involved in cancer 30-32 was a potential target of DSCAM-AS1. Recently studies showed that miR-137 might participate in the progression of CRC by directly targeting at EZH2 32, TCF4 31 and YBX1 33. Study showed that DSCAM-AS1 may act as a competing endogenous RNA of miR-137 and regulates EPS8 to promote cell reproduction and suppresses cell apoptosis in TR breast cancer 34. RT-qPCR results in our study displayed that the expression of miR-137 in CRC tissues was relatively lower than that in normal tissues, and was negatively correlated with the expression of DSCAM-AS1. The luciferase reporter assay further proved that miR-137 was a direct target of DSCAM-AS1.

Previous studies have reported that miR-137 could directly target on Notch1 and participate in diabetic kidney disease 35, ischemic stroke 36, and hypoxia-induced retinal ganglion cell apoptosis 37. Our finding indicated that silence of DSCAM-AS1 and miR-137 mimics could suppress Notch-1 signaling, which is a novel pathway regulating cell proliferation and EMT. Moreover, the results of our study suggested that miR-137 can directly worked on the 3'-UTR of Notch-1, thus suppress its expression. To further explore the association between DSCAM-AS1, miR-137 and Notch-1, rescued-function test was used, and found that DSCAM-AS1 might elicit promoting impact on CRC cell proliferation and migration via miR-137/Notch-1 axis. Recently, it was also reported that DSCAM-AS1 might promote the migration and invasion of colorectal cells by regulating miR-216b 25. However, this previous research failed to figure out the specific target mRNA to form a complete lncRNA-miRNA-mRNA axis. MiR-137 is a novel microRNA involved in colorectal cancer 30, 38 and Notch-1 plays an important role in promoting the progression of CRC 39. Our study proved that miR-137 directly targets at Notch-1 by binding with its 3'-UTR, and DSCAM-AS1 regulates the miR-137/Notch-1 axis.

In summary, our findings revealed the role of DSCAM-AS1/miR-137/Notch-1 axis in the progression of CRC. DSCAM-AS1 may serve as a promising factor to predict prognosis and therapeutic target against CRC.

## Figures and Tables

**Table 1:** Correlation between clinical characteristics and DSCAM-AS1 expression in patients with CRC

Characteristic	DSCAM-AS1		*P*-value
	Low	High	
Gender			n.s.
Male	11	15	
Female	14	11	
Age	63.1±2.8	62.1±2.5	n.s.
Location			n.s.
Colon	19	20	
Rectal	6	6	
TNM stage			<0.05
I-II	17	7	
III-IV	8	19	
Metastasis			<0.05
Yes	9	20	
No	16	6	
Differentiation			n.s.
Well	6	5	
Moderate	13	14	
Poor	5	7	

n.s.: no significance

## Abbreviations

lncRNAs: long noncoding RNAs
CRC: colorectal cancer
ceRNA: competing endogenous RNA
miRNAs: microRNAs
FBS: fetal bovine serum
EMT: Epithelial to mesenchymal transition
TNM: Tumor, Node, Metastasis
UICC: International Union against Cancer

## References

[1] Torre LA, Bray F, Siegel RL, Ferlay J, Lortet-Tieulent J, Jemal A (2015). Global cancer statistics, 2012. CA: a cancer journal for clinicians.

[2] Arnold M, Sierra MS, Laversanne M, Soerjomataram I, Jemal A, Bray F (2017). Global patterns and trends in colorectal cancer incidence and mortality. Gut.

[3] Siegel R, Naishadham D, Jemal A (2012). Cancer statistics, 2012. CA: a cancer journal for clinicians.

[4] Manfredi S, Lepage C, Hatem C, Coatmeur O, Faivre J, Bouvier AM (2006). Epidemiology and management of liver metastases from colorectal cancer. Annals of surgery.

[5] Wilusz JE, Sunwoo H, Spector DL (2009). Long noncoding RNAs: functional surprises from the RNA world. Genes & development.

[6] Schmitz SU, Grote P, Herrmann BG (2016). Mechanisms of long noncoding RNA function in development and disease. Cellular and molecular life sciences: CMLS.

[7] Sun S, Gong C, Yuan K (2019). LncRNA UCA1 promotes cell proliferation, invasion and migration of laryngeal squamous cell carcinoma cells by activating Wnt/beta-catenin signaling pathway. Experimental and therapeutic medicine.

[8] Lin WC, Yan MD, Yu PN, Li HJ, Kuo CC, Hsu CL (2013). The role of Sp1 and EZH2 in the regulation of LMX1A in cervical cancer cells. Biochimica et biophysica acta.

[9] Zhu C, Huang L, Xu F, Li P, Li P, Hu F (2020). LncRNA PCAT6 promotes tumor progression in osteosarcoma via activation of TGF-beta pathway by sponging miR-185-5p. Biochemical and biophysical research communications.

[10] Liang WC, Fu WM, Wong CW, Wang Y, Wang WM, Hu GX (2015). The lncRNA H19 promotes epithelial to mesenchymal transition by functioning as miRNA sponges in colorectal cancer. Oncotarget.

[11] Yoon JH, Abdelmohsen K, Srikantan S, Yang X, Martindale JL, De S (2012). LincRNA-p21 suppresses target mRNA translation. Molecular cell.

[12] Kallen AN, Zhou XB, Xu J, Qiao C, Ma J, Yan L (2013). The imprinted H19 lncRNA antagonizes let-7 microRNAs. Molecular cell.

[13] Liang WH, Li N, Yuan ZQ, Qian XL, Wang ZH (2019). DSCAM-AS1 promotes tumor growth of breast cancer by reducing miR-204-5p and up-regulating RRM2. Molecular carcinogenesis.

[14] Liao J, Xie N (2019). Long noncoding RNA DSCAM-AS1 functions as an oncogene in non-small cell lung cancer by targeting BCL11A. European review for medical and pharmacological sciences.

[15] Li Y, Hao J, Jiang YM, Liu Y, Zhang SH (2019). Long non-coding RNA DSCAM-AS1 indicates a poor prognosis and modulates cell proliferation, migration and invasion in ovarian cancer via upregulating SOX4. European review for medical and pharmacological sciences.

[16] Sun W, Li AQ, Zhou P, Jiang YZ, Jin X, Liu YR (2018). DSCAM-AS1 regulates the G1 /S cell cycle transition and is an independent prognostic factor of poor survival in luminal breast cancer patients treated with endocrine therapy. Cancer medicine.

[17] Huang YL, Xu Q, Wang X (2019). Long noncoding RNA DSCAM-AS1 is associated with poor clinical prognosis and contributes to melanoma development by sponging miR-136. European review for medical and pharmacological sciences.

[18] Yan Y, Su M, Qin B (2020). CircHIPK3 promotes colorectal cancer cells proliferation and metastasis via modulating of miR-1207-5p/FMNL2 signal. Biochemical and biophysical research communications.

[19] Feng L, He M, Rao M, Diao J, Zhu Y (2019). Long noncoding RNA DLEU1 aggravates glioma progression via the miR-421/MEF2D axis. OncoTargets and therapy.

[20] Niknafs YS, Han S, Ma T, Speers C, Zhang C, Wilder-Romans K (2016). The lncRNA landscape of breast cancer reveals a role for DSCAM-AS1 in breast cancer progression. Nature communications.

[21] Chu JYS, Chau MKM, Chan CCY, Tai ACP, Cheung KF, Chan TM (2019). miR-200c Prevents TGF-beta1-Induced Epithelial-to-Mesenchymal Transition and Fibrogenesis in Mesothelial Cells by Targeting ZEB2 and Notch1. Molecular therapy Nucleic acids.

[22] Baker A, Wyatt D, Bocchetta M, Li J, Filipovic A, Green A (2018). Notch-1-PTEN-ERK1/2 signaling axis promotes HER2+ breast cancer cell proliferation and stem cell survival. Oncogene.

[23] Khorshidi H, Azari I, Oskooei VK, Taheri M, Ghafouri-Fard S (2019). DSCAM-AS1 up-regulation in invasive ductal carcinoma of breast and assessment of its potential as a diagnostic biomarker. Breast disease.

[24] Ji D, Hu G, Zhang X, Yu T, Yang J (2019). Long non-coding RNA DSCAM-AS1 accelerates the progression of hepatocellular carcinoma via sponging miR-338-3p. American journal of translational research.

[25] Liu F, Jia J, Sun L, Yu Q, Duan H, Jiao D (2019). lncRNA DSCAM-AS1 downregulates miR-216b to promote the migration and invasion of colorectal adenocarcinoma cells. OncoTargets and therapy.

[26] Salmena L, Poliseno L, Tay Y, Kats L, Pandolfi PP (2011). A ceRNA hypothesis: the Rosetta Stone of a hidden RNA language?. Cell.

[27] Lin M, Li Y, Xian J, Chen J, Feng Y, Mao C (2020). Long non-coding RNA AGER-1 inhibits colorectal cancer progression through sponging miR-182. The International journal of biological markers.

[28] Zheng S, Lin F, Zhang M, Fu J, Ge X, Mu N (2019). AK001058 promotes the proliferation and migration of colorectal cancer cells by regulating methylation of ADAMTS12. American journal of translational research.

[29] Liu Y, Zhou J, Wang S, Song Y, Zhou J, Ren F (2019). Long non-coding RNA SNHG12 promotes proliferation and invasion of colorectal cancer cells by acting as a molecular sponge of microRNA-16. Experimental and therapeutic medicine.

[30] Fu Y, Yin Y, Peng S, Yang G, Yu Y, Guo C (2019). Small nucleolar RNA host gene 1 promotes development and progression of colorectal cancer through negative regulation of miR-137. Molecular carcinogenesis.

[31] Bi WP, Xia M, Wang XJ (2018). miR-137 suppresses proliferation, migration and invasion of colon cancer cell lines by targeting TCF4. Oncology letters.

[32] Liu X, Cui L, Hua D (2018). Long Noncoding RNA XIST Regulates miR-137-EZH2 Axis to Promote Tumor Metastasis in Colorectal Cancer. Oncology research.

[33] Guo Y, Pang Y, Gao X, Zhao M, Zhang X, Zhang H (2017). MicroRNA-137 chemosensitizes colon cancer cells to the chemotherapeutic drug oxaliplatin (OXA) by targeting YBX1. Cancer biomarkers: section A of Disease markers.

[34] Ma Y, Bu D, Long J, Chai W, Dong J (2019). LncRNA DSCAM-AS1 acts as a sponge of miR-137 to enhance Tamoxifen resistance in breast cancer. Journal of cellular physiology.

[35] Han F, Wang S, Chang Y, Li C, Yang J, Han Z (2018). Triptolide prevents extracellular matrix accumulation in experimental diabetic kidney disease by targeting microRNA-137/Notch1 pathway. Journal of cellular physiology.

[36] Chen F, Zhang L, Wang E, Zhang C, Li X (2018). LncRNA GAS5 regulates ischemic stroke as a competing endogenous RNA for miR-137 to regulate the Notch1 signaling pathway. Biochemical and biophysical research communications.

[37] Li H, Zhu Z, Liu J, Wang J, Qu C (2018). MicroRNA-137 regulates hypoxia-induced retinal ganglion cell apoptosis through Notch1. International journal of molecular medicine.

[38] Sun Y, Zhao X, Zhou Y, Hu Y (2012). miR-124, miR-137 and miR-340 regulate colorectal cancer growth via inhibition of the Warburg effect. Oncology reports.

[39] Lan G, Lin Z, Zhang J, Liu L, Zhang J, Zheng L (2019). Notch pathway is involved in the suppression of colorectal cancer by embryonic stem cell microenvironment. OncoTargets and therapy.

